# Supplementary material for: Population size interacts with reproductive longevity to shape the germline mutation rate
Source: bioRxiv. 2024 Nov 9:2023.12.06.570457. Preprint. [Version 2] doi: 10.1101/2023.12.06.570457 (PMC11580940; doi:10.1101/2023.12.06.570457)
Supplement: Supplement 2 [file NIHPP2023.12.06.570457v2-supplement-2.pdf]

bioRxiv preprint doi: https://doi.org/10.1101/2023.12.06.570457; this version posted November 9, 2024. The copyright holder for this preprint (which was not certified by peer review) is the author/funder, who has granted bioRxiv a license to display the preprint in perpetuity. It is made available under aCC-BY 4.0 International license.

Supplementary Figures

bioRxiv preprint doi: https://doi.org/10.1101/2023.12.06.570457; this version posted November 9, 2024. The copyright holder for this preprint (which was not certified by peer review) is the author/funder, who has granted bioRxiv a license to display the preprint in perpetuity. It is made available under aCC-BY 4.0 International license.

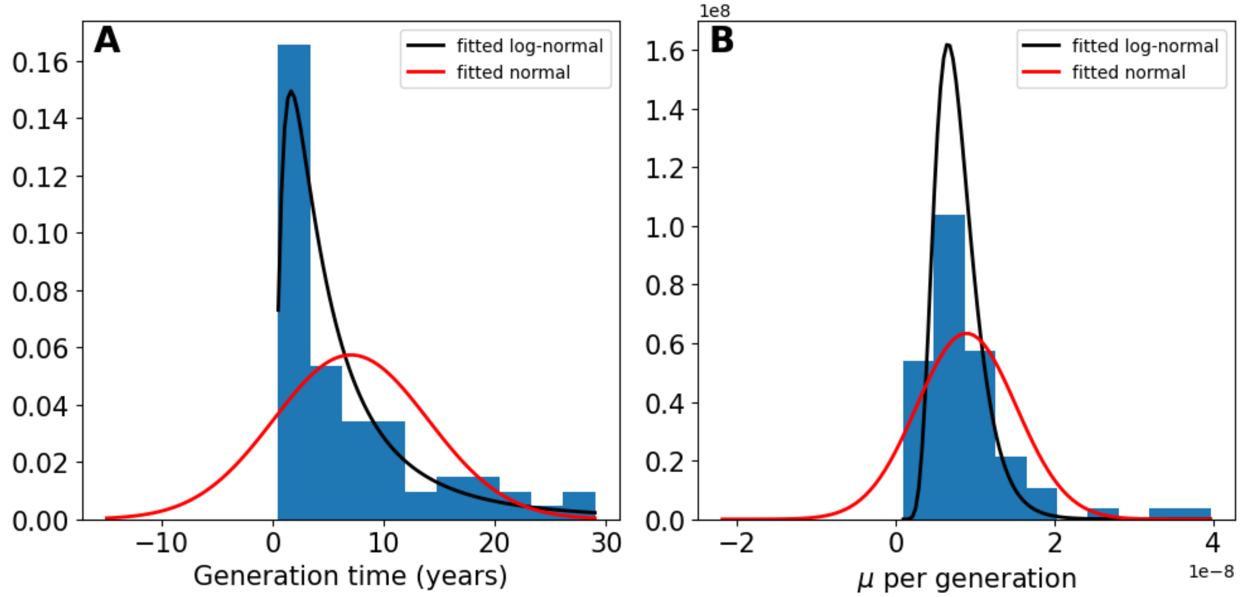

Supplementary Figure 1: Distributions of generation time and mutation rate per generation across species. Data taken from Wang and Obbard (26). Red and black lines correspond to the fitted normal and log-normal distributions, respectively. Lognormal provides a better fit to both the distribution of generation times and the distribution of the mutation rate per generation.

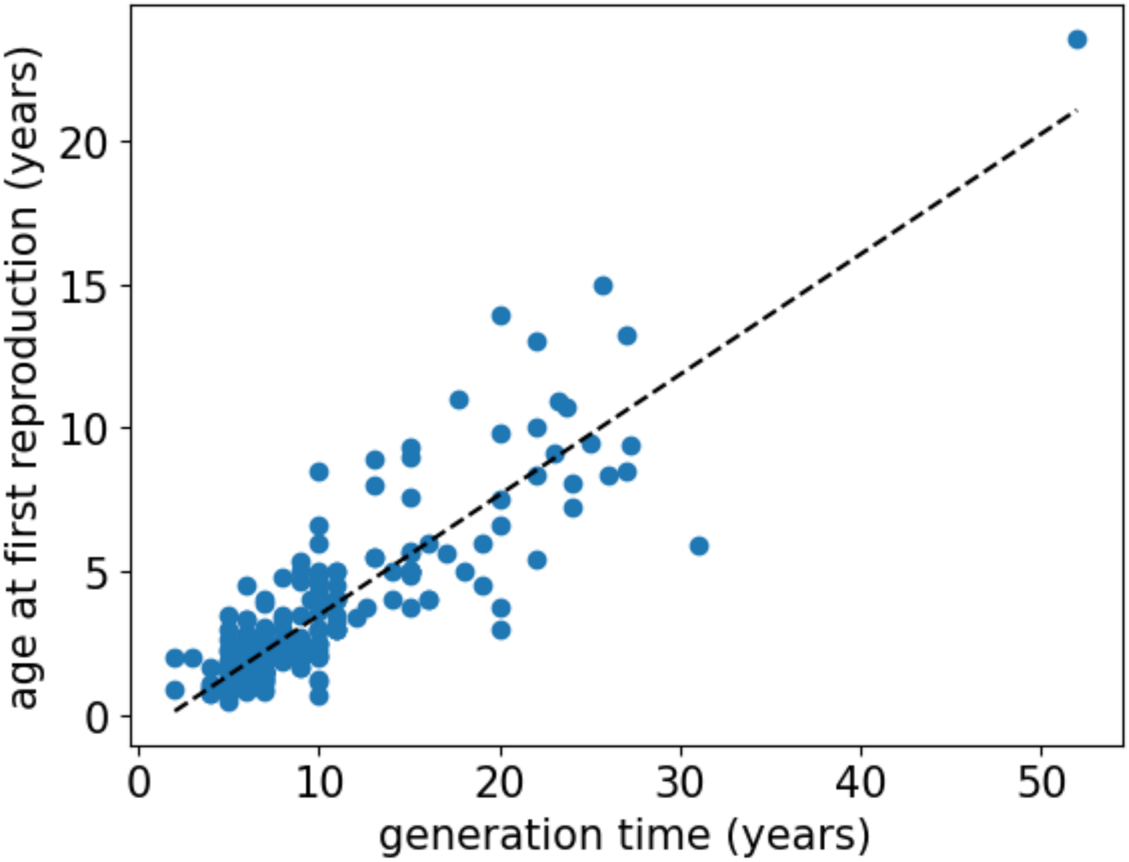

bioRxiv preprint doi: https://doi.org/10.1101/2023.12.06.570457; this version posted November 9, 2024. The copyright holder for this preprint (which was not certified by peer review) is the author/funder, who has granted bioRxiv a license to display the preprint in perpetuity. It is made available under aCC-BY 4.0 International license.

Supplementary Figure 2: Regression of age at first reproduction versus generation time. Data taken from Pacifici et al. (52). Age at first reproduction is used as a proxy for age at puberty. Age at first reproduction is found to be linear with respect to generation time, with a slope of 0.42.
